# Giant Second Harmonic Generation Enhancement by Ag Nanoparticles Compactly Distributed on Hexagonal Arrangements

**DOI:** 10.3390/nano11092394

**Published:** 2021-09-14

**Authors:** Alejandro Gómez-Tornero, Luisa E. Bausá, Mariola O. Ramírez

**Affiliations:** Departamento Física de Materiales, Instituto de Materiales Nicolás Cabrera and Condensed Matter Physics Center (IFIMAC), Universidad Autónoma de Madrid, 28049 Madrid, Spain; alejandrogomeztornero@gmail.com (A.G.-T.); luisa.bausa@uam.es (L.E.B.)

**Keywords:** metallic nanoparticles, second harmonic generation, hybrid plasmonic-dielectric, nanophotonics, nonlinear optics, hexagonal microcavity, lithium niobate

## Abstract

The association of plasmonic nanostructures with nonlinear dielectric systems has been shown to provide useful platforms for boosting frequency conversion processes at metal-dielectric interfaces. Here, we report on an efficient route for engineering light–matter interaction processes in hybrid plasmonic-χ^(2)^ dielectric systems to enhance second harmonic generation (SHG) processes confined in small spatial regions. By means of ferroelectric lithography, we have fabricated scalable micrometric arrangements of interacting silver nanoparticles compactly distributed on hexagonal regions. The fabricated polygonal microstructures support both localized and extended plasmonic modes, providing large spatial regions of field enhancement at the optical frequencies involved in the SHG process. We experimentally demonstrate that the resonant excitation of the plasmonic modes supported by the Ag nanoparticle-filled hexagons in the near infrared region produces an extraordinary 10^4^-fold enhancement of the blue second harmonic intensity generated in the surface of a LiNbO_3_ crystal. The results open new perspectives for the design of efficient hybrid plasmonic frequency converters in miniaturized devices.

## 1. Introduction

The generation and manipulation of nonlinear optical processes at the nanoscale is a highly interesting field due to its broad range of applications in a variety of disciplines such as biology, physics, chemistry, materials science, and information technologies [1,2,3]. However, limitations associated with the low conversion efficiency at small scales require the use of novel designs to develop efficient nonlinear optical sources operating in extremely confined volumes. In the last decade, different approaches based on plasmon-driven light confinement have been used to enhance the nonlinear response of a variety of systems of reduced dimensions. In fact, the large near field enhancement provided by localized surface plasmon resonances (LSPRs) compensates for the lack of phase matching mechanisms at the nanoscale, leading to high nonlinear responses at subwavelength scales [4,5,6]. Depending on the size and geometry of the plasmonic nanostructures, the enhancement of the electromagnetic field can be achieved either at the harmonic or the fundamental frequency, the latter being much more efficient due to the nonlinear dependence of the generated harmonic signal with the fundamental radiation [7,8,9,10].

In recent years, the use of plasmonic nanostructures for light confinement in nonlinear dielectrics has emerged as an efficient and straightforward approach for boosting the frequency doubling response in miniaturized components. This approach exploits both the high nonlinear coefficients offered by certain nonlinear dielectric crystals and the strong light confinement provided by plasmonic structures, which has led to efficient hybrid plasmonic-χ^(2)^ dielectric systems operating at different spectral ranges. The proposed designs include core-shell nanoparticles (ferroelectric nanoparticles surrounded by Au shells [11,12]), semiconductors or nonlinear dielectrics in the gap between plasmonic nanoantennas [13,14,15], colloidal BTO-Au hybrid nano-dimmers [16], Au nanoring resonators filled with LiNbO_3_ [17], hybrid plasmonic waveguides [18,19], or ordered and disordered plasmonic arrangements of interacting silver nanoparticles (NPs) assembled on the polar surface of ferroelectric crystals [20,21,22,23,24].

In this work, we use an alternative approach to demonstrate SHG enhancement by around four orders of magnitude in LiNbO_3_ by means of interacting Ag NPs compactly distributed within polygonal surfaces onto a ferroelectric domain patterned nonlinear LiNbO_3_ crystal. LiNbO_3_ is one of the most widely employed dielectrics for advanced photonics and optoelectronics due to its remarkable acousto-optic, electro-optic, and nonlinear coefficients [25,26,27]. The association of LiNbO_3_ with noble metal plasmonic nanostructures has shown to be a useful route to provide solid-state platforms for boosting nonlinear processes at the nanoscale [17,19,21,28]. SHG enhancement factors as high as 400 have been reported by using hexagonal plasmonic necklaces of silver nanoparticles formed on the LiNbO_3_ crystal surface [21]. Additionally, theoretical works have pointed out the possibility of achieving giant SHG enhancements by using either Ag-coated LiNbO_3_ core-shell nanocuboids with designed double plasmonic resonances [29] or hybrid nanoplasmonic waveguides [30].

Here, we go a step further to experimentally demonstrate that SHG in LiNbO_3_ can be enhanced by around four orders of magnitude by completely filling the inside of the hexagonal necklaces with interacting silver NPs. The fabricated plasmonic structure provides two spectrally overlapping plasmonic resonances; namely, a collective localized plasmonic mode sustained by the surrounding hexagonal polygonal necklace (centered at around 535 nm) and a spatially extended plasmonic mode centered at 705 nm, which is associated with the LSPR of the Ag NPs covering the hexagonal regions. These Ag Np filled hexagons are responsible for an efficient enhancement of the fundamental NIR radiation, thus leading to giant SHG enhancement values of about 10^4^ in the blue spectral region due to the two-photon character of the nonlinear process. The results provide an alternative strategy to design cost-efficient and scalable plasmonic architectures to enhance SHG processes in hybrid plasmonic-dielectric systems, opening new opportunities for increasing the nonlinear conversion efficiency in small volumes.

The manuscript is structured as follows. First, we briefly describe the fabrication procedure and the main sample features. Then, the optical response of large-filled hexagons (5–10 µm side length) as well as their capability to boost the SHG in the nonlinear material are studied and compared to those obtained for empty hexagonal necklaces with similar dimensions. Finally, the influence of the hexagon size on the SHG enhancement is analyzed. The results show that by reducing the size of the filled hexagons to dimensions close to the fundamental radiation wavelength (size length of hexagons around 700 nm), the nonlinear emission process is highly improved and an extraordinary 10^4^-fold enhancement of the blue second harmonic intensity generated in the surface of a LiNbO_3_ crystal is obtained. The results, related to the interconnection of the NPs structure and to the collective modes in the wavelength-scale hexagonal arrangements, validate the optical performance of the hybrid system in a spectral region of high technological interest.

## 2. Materials and Methods

### 2.1. Sample Preparation

Domain Fabrication: Two dimensional patterns of hexagonal ferroelectric domains were fabricated in a 0.5 mm thick z-cut LiNbO_3_ single domain crystal by direct electron beam irradiation using a Philips XL30 SFEG electron microscope driven by an Elphy Raith nanolithography software. The beam current and acceleration voltage were fixed at 0.3 nA and 15 kV, respectively. Additionally, during the irradiation process, the *z+* face was coated with an Al film of 100 nm acting as ground electrode. After irradiation, scanning electron microscopy (SEM) microscopy was employed to confirm that the inverted hexagonal domains crossed the whole sample thickness, their size and shape being similar at both crystal faces [31,32].

Ag Decoration: The photo-deposition of metallic NPs was carried out by ferroelectric lithography by immersing the domain patterned crystal into a 0.01 M AgNO_3_ solution at 60 °C while their polar surface was illuminated with above band gap UV radiation with a mercury pen lamp (UVP model 11SC-1) with its main line at 253.6 nm. The Hg lamp emission power was 5400 μW/cm^2^ at a distance of 2 cm. The illumination time was varied to achieved different types of hybrid structures. More specifically, the formation of empty hexagonal necklaces was achieved for an illumination time of 4 min and the fabrication of Ag-filled hexagonal regions was achieved after illuminating the AgNO_3_ solution for 15 min. In both cases, the photoinduced silver deposition process was carried out on optical grade polished crystals. Finally, it is worth mentioning that while the formation of hexagonal necklaces can be achieved illuminating the crystal at any polar face, the Ag-filled hexagonal regions correspond to ferroelectric domains with a *z+* positive polarity. That is, the photoinduced silver deposition must be carried out by illuminating the *z−* face of the crystal containing *z+* hexagonal domains.

### 2.2. Optical Characterization

Dark field images were obtained in transmission configuration by using an Olympus BX51 microscope equipped with a dark-field condenser. For the extinction spectra, a double beam Lambda 1050 PerkinElmer spectrometer was employed. 

Spatially resolved SHG experiments were carried out by means of a laser scanning confocal microscope (Olympus BX41) provided with a two-axis XY motorized platform driven by commercial Labspec software. The spatial resolution of the stage was 0.3 μm. The fundamental beam, delivered by a femtosecond pulsed Ti:sapphire laser (Spectra Physics Model 177-Series), was tuned at 850 nm and focused onto the sample surface by a 100× microscope objective. The SHG signal was collected in backscattering geometry with the same objective and detected by a Peltier cooled photomultiplier tube. 

## 3. Results and Discussion

### 3.1. Fabrication Process. Sample Characteristics

Ag NPs assembled on hexagonal arrangements were obtained on the polar surface of LiNbO_3_ crystals by using hexagonal inverted ferroelectric domains as artificial templates for selective photo-deposition process [21,22]. The side length of the hexagons varied from 0.7 µm up to 10 μm, in such a way that the contour of a single hexagon consisted of a necklace containing hundreds of Ag NPs. The domain selective formation of Ag NPs on a positive domain is based on the photo-chemical reactions that take place on the surface of domain patterned ferroelectric crystals [33,34]. More specifically, when the patterned ferroelectric is placed into an AgNO_3_ solution and illuminated with above-band-gap UV light, the screening charge produces electronic photocarriers at the crystal surface, which participate in the chemical reaction reducing the silver cations on the specific polarity domains [34]. In the case of LiNbO_3_, Ag NP formation involves both a weak downward band bending at positive domain surfaces and a strong electric field at domain boundaries [35]. This allows a certain degree of control over the process so that the Ag deposition on the domain surface and the deposition on the domain boundary surface can be achieve independently. Accordingly, by modifying the illumination time, either Ag NPs hexagonal necklaces formed on the domain wall boundaries or a dense distribution of closely spaced interacting Ag nanoparticle covering the positive domain surface can be obtained. Specific details on the preparation method can be found elsewhere [22].

**Figure 1:**
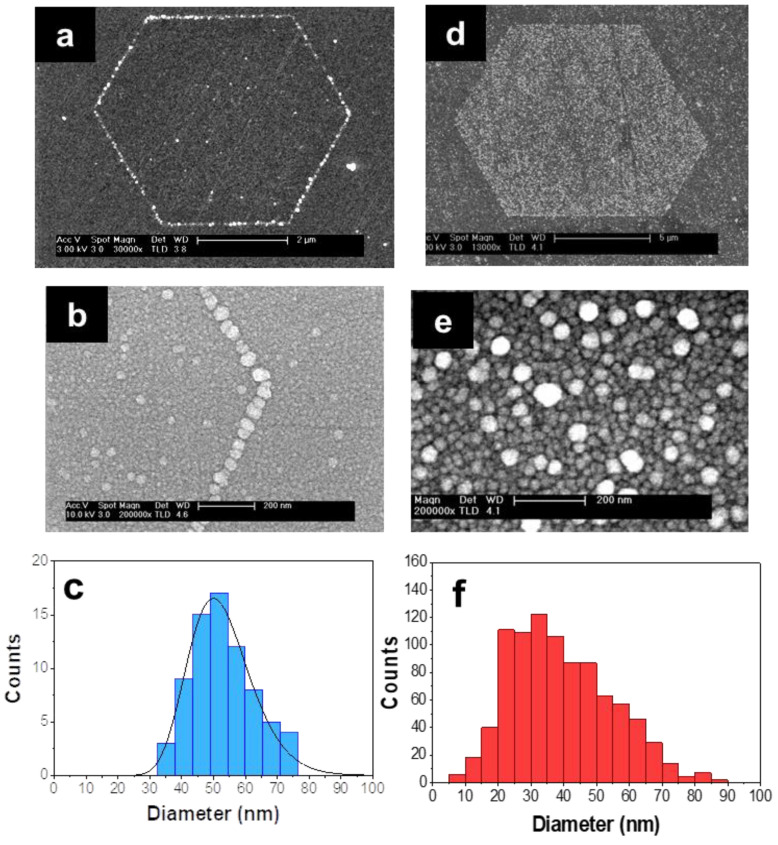
Plasmonic hexagonal arrangements on LiNbO_3_ substrate. SEM images of (**a**) Hexagonal plasmonic necklace formed on the domain boundary surface of LiNbO_3_ at a photo-deposition time of 4 min. (**b**) Detailed view of the Ag NPs forming the vertex of a hexagonal necklace. (**c**) Histogram of the Ag NPs diameter forming the necklace. (**d**) Filled hexagonal domain. It is formed by a dense distribution of Ag NPs covering the positive domain surface after a photo-deposition time of 15 min. (**e**) Detail of the Ag NPs that cover a positive ferroelectric domain of hexagonal shape. (**f**) Histogram of the Ag NPs diameter covering the filled hexagonal region.

Figure 1 shows SEM images of the resultant metallic nanostructures after different photo-deposition processes. By varying the illumination time while keeping constant the rest of the parameters (0.01 M AgNO_3_ solution, 5400 μW/cm^2^ UV emission power, 60 °C substrate temperature) different type of plasmonic arrangements are obtained on the polar surface of LiNbO_3_. Figure 1a shows a hexagonal plasmonic necklace of Ag NPs on the domain boundary surface of LiNbO_3_ obtained for an illumination time of 4 min. As observed, the chain of Ag NPs is perfectly located on the domain boundary surface of a hexagonal ferroelectric domain, bending at vertices to form a polygonal necklace (Figure 1b). According to the distribution displayed in Figure 1c, the average size of the Ag NPs forming the hexagonal necklace is around 50 nm and the interparticle distance about 2 nm.

Increasing the illumination time leads to the formation of Ag NPs that completely cover the surface of positive ferroelectric domains after 15 min of illumination. This assembling of nearby NPs within the hexagon provides a straightforward fabrication of large spatial regions of high density of interacting plasmonic NPs confined inside a specific polygonal zone (Figure 1d). A detailed image of the Ag NPs located inside the plasmonic hexagon is shown in Figure 1e. As can be seen, a high density of mostly spherical Ag NPs fills the polygonal region. Although in this case, a slightly larger size dispersion is obtained (Figure 1f), the average NP size is similar to that of the Ag NPs necklaces. Additionally, comparable values of the interparticle distance are obtained in both type of Ag NPs arrangements (around 2 nm). Finally, it is worth mentioning that the fabricated polygonal microstructures can be organized in two-dimensional superlattices leading to scalable functional nonlinear metasurfaces [36].

### 3.2. Optical Response of Filled Polygonal Assemblies

**Figure 2:**
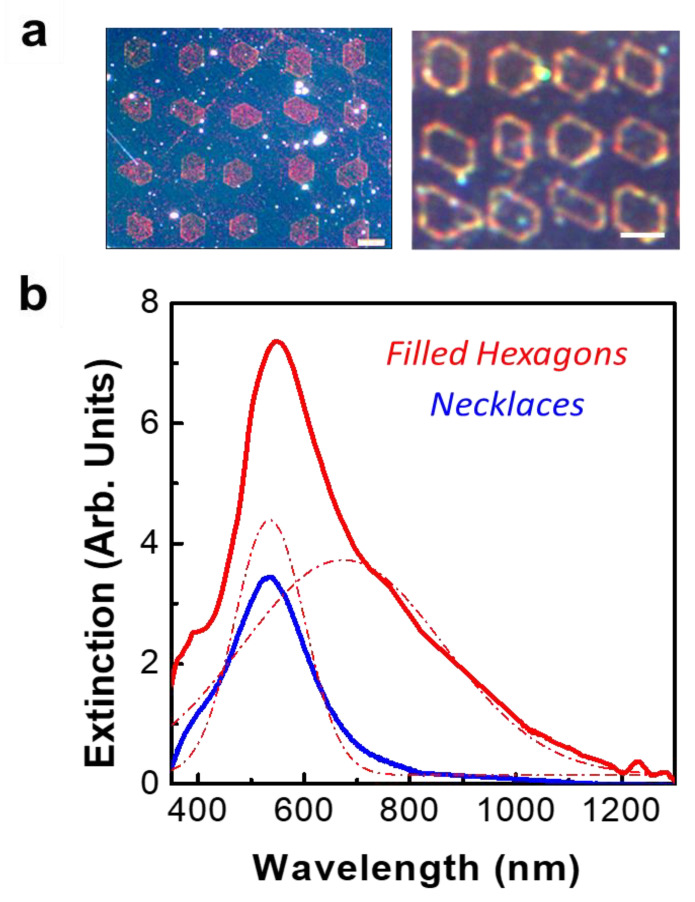
Far field optical response of the polygonal assemblies. (**a**) Dark-field scattering images recorded for the two types of Ag NPs hexagonal arrangement. Left: filled hexagons, Right: hexagonal necklaces. The scale bar is 5 μm. (**b**) Extinction spectra obtained in transmission mode after subtracting the contribution from the bare LiNbO_3_ substrate. Blue: hexagonal necklaces. Red: filled hexagons. The dashed red lines correspond to Gaussian fitting functions.

The dark-field scattering image of both types of arrangements is displayed in Figure 2. In both cases, the radiative nature of the plasmonic modes is observed. For the filled hexagons, a predominantly reddish color spreads throughout the decorated domain surface (Figure 2a). Differently, the dark field image associated with the hexagonal necklaces (short photo-deposition times) shows a dominant orange color, the scattered light being produced along the contour of the necklace where the Ag NPs are located. The corresponding extinction spectra obtained in transmission mode after subtracting the contribution from the bare LiNbO_3_ substrate are depicted in Figure 2b. As seen, in both cases a broad resonance with the maximum around λ_I_ = 535 nm is observed. In the case of the Ag NPs hexagonal necklaces, the spectrum corresponds to the collective mode sustained by the necklaces as previously reported by the authors [21,37]. In the case of the filled hexagons, the additional spectral band appearing at the low energy side (centered at around λ_II_ = 705 nm) can be related to the highly dense Ag NPs distribution decorating the interior of the hexagon. Both the λ_I_ and λ_II_ values correspond to the central wavelength of two Gaussian profiles that fit the extinction spectrum of the plasmonic structures shown in Figure 2b.

The appearance of the low energy band can be explained in the frame of previous studies, which show that complex NP structures can be efficiently analyzed by an embedded chain plasmon model [38,39,40]. In this context, the spectral response of the complex plasmonic structure filling the hexagons can be interpreted as the contribution of a variety of linear or quasi-linear kinked NP chains within the hexagons. As a result, numerous plasmonic chains, with different numbers of interacting NPs, contribute to the total spectral response in the far field. Moreover, significant chain coupling has been reported for chain separation distances of a few nanometers, which shifts the resonance to the near infrared region [41]. This chain plasmon mode has been shown to be useful to enhance a variety of optical phenomena including SHG, SERS or nanolasing, because of its robustness, strong radiative nature, and broad spectral response [21,42,43]. On the other hand, the assembly of NPs inside the hexagons also shows certain similarity to percolated systems or disordered plasmonic networks where a spectral shift of the plasmonic resonance towards the near infrared spectral region has been reported due to the presence of NP clusters extended in relatively large spatial regions [44,45,46].

**Figure 3:**
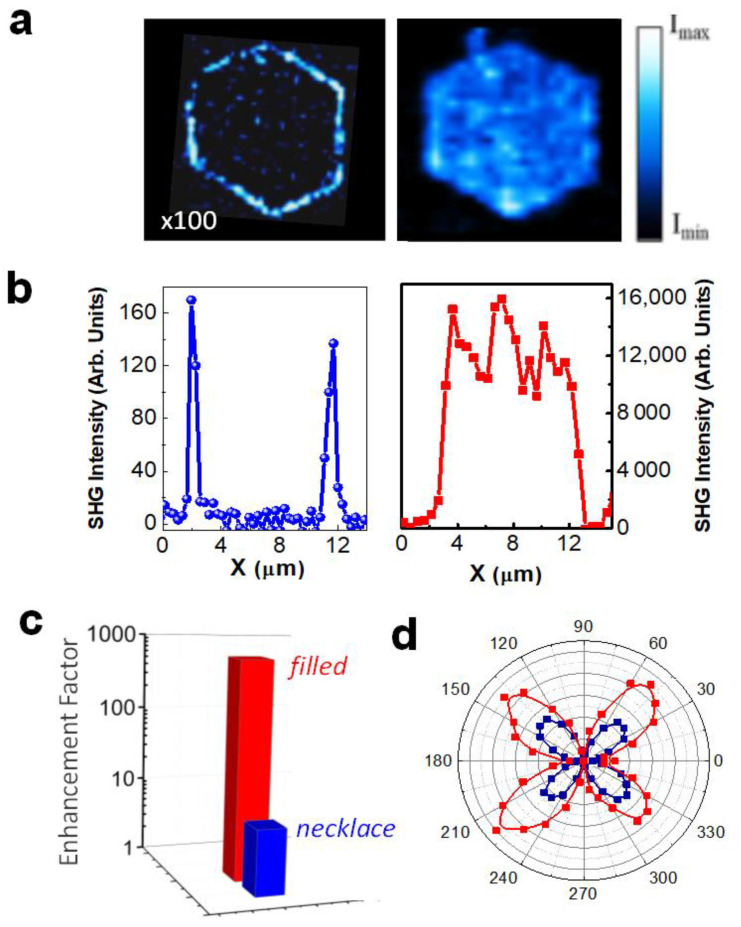
SHG enhancement: Filled hexagons vs hexagonal necklaces. (**a**) Spatial distribution of the SHG intensity generated in the LNbO_3_ substrate in the proximities of an Ag NPs hexagonal necklace (left) and Ag NPs filled hexagon (right) for a fundamental radiation wavelength of 850 nm. The SHG intensity map associated with the necklaces is multiplied by a factor of 100. The scale bar corresponds to 5 µm. (**b**) SHG intensity profile obtained from the SHG maps shown in (**a**). The fundamental pump power was about 1 mW. (**c**) Comparison between the SHG enhancement factor for the filled hexagon (red) and for the hexagonal necklace (blue). (**d**) Variation of the polarized SHG intensity as a function of the polarization angle of the incident radiation for filled hexagons (red) and bare LiNbO_3_ (dark blue).

The effect of the plasmonic arrangements on the SHG response generated by the nonlinear substrate was analyzed by means of confocal scanning microscopy. The experiments were performed at a fundamental wavelength of 850 nm, which efficiently overlaps the low energy side of the spectra shown Figure 2. The LiNbO_3_ substrate also exhibits a good transparency range for this wavelength, enabling the generation of SHG in the blue spectral region, which is of technological interest in the context of potential nonlinear photonic nanodevices. Figure 3 shows the spatial distribution of the blue SHG signal generated in the LiNbO_3_ substrate, which is enhanced by the plasmonic nanostructures. The spatial maps displayed in Figure 3a show that the SHG signal is greatly boosted in the presence of the metallic nanostructures with respect to that of the bare substrate, reproducing the spatial distribution of the Ag nanoparticles. That is, the SHG enhancement remains localized at the domain boundary surfaces in the case of hexagonal necklaces and extends over the whole domain surface in the case of the Ag NP filled domains. For illustrative purposes, the SHG spatial maps were normalized to their maximum values. The cross-section intensity profiles depicted in Figure 3b show the quantitative comparison of the SHG enhancement in both systems. As can be seen, the enhanced SHG intensity shows remarkable differences when comparing the response of both types of structures, the improved nonlinear response in the filled hexagons being two orders of magnitude greater than that obtained from the necklaces.

To estimate the enhancement factor, we compared the nonlinear performance of the hybrid architectures with respect to that generated in the substrate in the absence of metallic NPs. To that end, we experimentally determined the fundamental pump power required to achieve a comparable SHG intensity. The results obtained for the three analyzed systems were: 26 mW for the bare LiNbO_3_ substrate, 3 mW for the hybrid LiNbO_3_-plasmonic necklaces, and 1 mW for the filled hexagons. Hence, according to the quadratic dependence of the SHG response on the fundamental power, an SHG enhancement value of 675 is obtained for the filled hexagons, while an enhancement factor of 9 is achieved for the hexagonal necklaces. Figure 3c compares in log scale the enhancement factors obtained from both types of plasmonic structures. The dramatic difference between them can be accounted for by the presence of the low energy plasmonic resonance associated with the filled hexagons (see Figure 2), which provides a much more efficient spectral overlap of the fundamental radiation with respect to the case of the hexagonal necklaces. Accordingly, a significantly larger SHG enhancement due to the two-photon character of the frequency conversion process is obtained. 

Finally, we should emphasize that the SHG signal is generated in the LiNbO_3_ nonlinear substrate. This fact was confirmed by analyzing the SHG intensity as a function of the polarization of the fundamental radiation. The resulting polar plots of SHG intensity for the bare substrate and for the Ag NPs filled hexagon obtained in reflection geometry are shown in Figure 3d. For the sake of comparison, the nonlinear signal in the absence of Ag NPs was registered with an incident pump power 20 times greater than the one employed for the filled hexagon (~1 mW). As observed, they show similar trends and both polar plots are consistent with the 3 m point group symmetry of LiNbO_3_. Therefore, we can conclude that the SH signal is generated in the nonlinear LiNbO_3_ crystal, the contribution of the Ag nanostructures to the SHG being negligible.

### 3.3. Scaling Effects on SHG Enhancement

**Figure 4:**
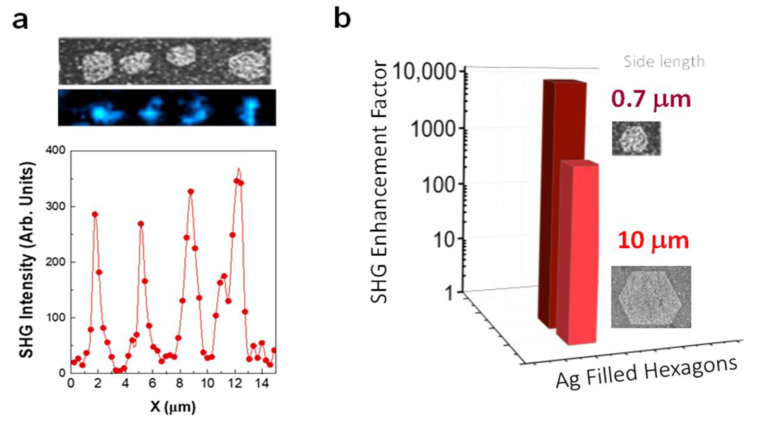
SHG enhancement by small-filled hexagons. (**a**) Top panel: SEM image and SHG spatial map from a region comprising four hexagons (hexagon sides around 700 nm). The blue regions in the map correspond to the SHG intensity. Bottom panel: SHG intensity area across the hexagons. (**b**) SHG enhancement factor for Ag NPs filled hexagons with hexagon sides of about 10 μm (red) and 700 nm (dark red).

Once the capability of large-filled hexagons to enhance the SHG process has been demonstrated, we aim to study the effect of reducing the hexagon size to dimensions comparable to that of the fundamental radiation wavelength. Figure 4a illustrates the SHG obtained for a distribution of hexagonal domains with side lengths around 700 nm. A striking difference is observed when comparing the enhancement factors obtained for large and small hexagons (Figure 4b). In contrast to the 670-fold value featured by the large-filled hybrid hexagons, the enhancement factor obtained for small hexagons is close to 10^4^. Indeed, the SHG spatial maps in these structures were recorded with an incident pump power of about 0.15 mW, that is one order of magnitude lower than that used for the large hexagons. 

The much larger enhancement in the small hexagons could be related to the existence of extended collective plasmonic modes propagating through the entire hexagon. In fact, the possibility of using the inner NP structure as plasmonic interconnection between the different sides of the hexagon could also favor the propagation of the plasmon mode inside the hexagon [47,48,49,50,51]. Additionally, due to the hexagon dimensions, the presence of modes in the wavelength-scale hexagonal nanocavity could also contribute to the light confinement and enhanced light–matter interaction process [52].

## 4. Summary and Conclusions

To summarize, we have presented an alternative hybrid plasmonic-nonlinear dielectric system for enhancing SHG processes at reduced dimensions. Using hexagonal plasmonic necklaces filled with a dense distribution of interacting Ag NPs, we show that the second harmonic response generated from the surface of a LiNbO_3_ crystal can be enhanced in about four orders of magnitude. Such a large enhancement is achieved by taking advantage of the strong near field generated at the metal-dielectric interface associated with the resonant excitation of localized and spatially extended plasmonic modes in small filled hexagons. The ability to combine both plasmonic fields in a single microstructure and the possibility to resonantly excite them by means of the optical fields involved in the nonlinear process, provides an efficient route for engineering light–matter interaction processes at subwavelength scales to enhance parametric nonlinear processes. Because of the ease of the fabrication process and the extraordinary improvement of the SHG response in small-confined region, our findings offer an alternative approach for the design of efficient hybrid plasmonic frequency converter nanodevices and opens interesting perspectives for a range of applications including sensing, imaging, information technologies, or future integrated circuits.

## Data Availability

Data underlying the results presented in this paper are not publicly available at this time but may be obtained from the authors upon reasonable request.
